# A Solution for Universal Classification of Species Based on Genomic DNA

**DOI:** 10.1155/2007/27894

**Published:** 2007-02-15

**Authors:** Mariko Kouduka, Daisuke Sato, Manabu Komori, Motohiro Kikuchi, Kiyoshi Miyamoto, Akinori Kosaku, Mohammed Naimuddin, Atsushi Matsuoka, Koichi Nishigaki

**Affiliations:** ^1^Department of Functional Materials Science, Saitama University, Saitama, Japan; ^2^Chitose Salmon Aquarium Chitose, Youth Educational Foundation, Chitose, Hokkaido, Japan; ^3^Institute of Medical Science, Dokkyo Medical University, Tochigi, Japan; ^4^Biol. Res. and Functions, National Inst. AIST, Tsukuba, Ibaraki, Japan; ^5^Department of Geology, Niigata University, Niigata, Japan; ^6^Rational Evolutionary Design of Advanced Biomolecules, Saitama Small Enterprise Promotion Corporation, SKIP City, Saitama, Japan

## Abstract

Traditionally, organisms have been classified on the basis of their phenotype. Recently, genotype-based classification has become possible through the development of sequencing technology. However, it is still difficult to apply sequencing approaches to the analysis of a large number of species due to the cost and labor. In most biological fields, the analysis of complex systems comprising various species has become an important theme, demanding an effective method for handling a vast number of species. In this paper, we have demonstrated, using plants, fish, and insects, that genome profiling, a compact technology for genome analysis, can classify organisms universally. Surprisingly, in all three of the domains of organisms tested, the phylogenetic trees generated from the phenotype topologically matched completely those generated from the genotype. Furthermore, a single probe was sufficient for the genome profiling, thereby demonstrating that this methodology is universal and compact.

## 1. INTRODUCTION

Because the functional and morphological diversities of an organism represent the value of the organism itself, the traditional biological techniques used to characterize these properties provide indispensable information. Conventional biology techniques face difficulties, however, such as classifying characterless organisms like microbes [1] and analyzing communities composed of huge numbers of various organisms [2], owing to both the instability of phenotypes, which are easily affected by environmental factors [3], and an insufficient number of experts [4].

A potential solution to these problems has been to characterize an organism according to the sequence of the small subunit ribosomal RNA (16S/18S rRNA), an approach that has been applied to various organisms [5–7]. Similarly, cytochrome oxygenase subunit 1 (COD1), gyrase, and other genes have been used for this purpose [8]. The superiority of these approaches is that they are based on popular and well-established sequencing technology and can provide the determinate result of nucleotide sequence, which can be further computer-analyzed and can fuel the activity of bioinformatics. Nevertheless, this approach cannot be said to be a readily usable method for classifying species because (i) it is rather costly and time-consuming for application to a large number of species (e.g., >100), especially for scientists in general all over the world, and (ii) it often results in an insufficient amount of information for identifying and classifying species [8]. The latter problem can be overcome by sequencing additional genes [8–10]; however, this makes the approach more complicated and less accessible.

Here, we present a solution for the universal classification of species together with a demonstration of its effectiveness, which has been tested by applying it to taxonomically well-established organisms such as plants, fish, and insects. Genome profiling (GP) has already been established as a method for the identification of species [11], and has sometimes been applied to clustering of organisms [12]. Due to the nature of the samples used in GP (mostly, bacteria, fungi, and protozoa, which taxonomically can sometimes be subject to debate), it has not been possible to establish GP as a technique for classification up till now. However, here we show for the first time that GP [13, 14] is successfully applied to the purpose of classification. Owing to its convenience and its highly informative nature, this technique of classification by GP can be widely applied to biological research, including botanical research.

## 2. MATERIALS AND METHODS

### 2.1. Materials

The plant, insect, and fish species used in this study are listed in Table 1.

### 2.2. DNA preparation

All samples were well washed with distilled water prior to DNA extraction. In particular, the legs of insects were vigorously washed with SDS detergent to remove contaminating microbes. DNAs of plants were prepared according to the conventional alkaline extraction method [16], whereas those of fish and insects were extracted by the phenol-chloroform-proteinase *K*-method [17] using a tiny portion (∼0.05 mg) of caudal fins or legs. For convenience, here we define genomic DNA as the whole set of DNAs contained in cell [13].

### 2.3. Genome profiling

GP is a well-established method [18] comprising the three following major steps: random PCR [19], temperature gradient gel electrophoresis (TGGE), [20] and data-normalization by computer-processing [18]. Random PCR has the ability to pick up, for example, the top ten DNA fragments that are generated by more stable hybridization of the primer DNA. The random PCR solution (50 *μ*l) contained 200 *μ*M dNTPs (N = G, A, T, C), 0.5 *μ*M primer, 10 mM Tris-HCl (pH 9.0), 50 mM KCl, 2.5 mM MgCl_2_, 0.02 unit/*μ*l Taq DNA polymerase (Takara Bio, Japan) and template DNA (arbitrary amount). Random PCR was carried out with 30 cycles of denaturation (94°C, 30 s), annealing (28°C, 2 min) and extension (47°C, 2 min) using a PTC-100TM PCR machine (MJ Research, Inc, USA). DNA samples, together with the internal reference DNA, were subjected to *μ*TGGE (micro-temperature gradient gel electrophoresis) [21] (one inch square size) using a *μ*-TG apparatus (Taitec, Japan) with a linear temperature gradient of 15°C to 60°C. The gel used was 6% acrylamide (acrylamide: bis = 19 : 1) containing 90 mM Tris-HCl, 90 mM boric acid, 2 mM EDTA (pH 8.0), and 8 M urea. Detection of DNA bands was carried out either by monitoring fluorescence with a fluorescence imager, Molecular Imager FX (BioRad, USA), or by staining with silver [12]. From the resulting band patterns, which were rather complicated, characteristic or “featuring” points (e.g., kinked points) were extracted and then processed with the aid of computer to generate *spiddos* (species identification dots) [11, 18]. Sets of *spiddos* are able to provide a sufficient amount of information for provisionally identifying species, which is usually done by calculating the pattern similarity score (PaSS) between two genomes [18]. Using *spiddos*, we can define PaSS of the genomes between two species as follows:

(1) $$\text{paSS}=1-\frac{1}{n}\sum_{i=1}^{n}\frac{\left|{\vec{P}}_{i}-{\vec{P}}_{i}^{\prime }\right|}{\left|{\vec{P}}_{i}|+|{\vec{P}}_{i}^{\prime }\right|}$$, 0 ≤ PaSS ≤ 1,

where ${\vec{P}}_{i}$ and ${\vec{P}}_{i}^{\prime }$ correspond to the normalized positional vectors (composed of two elements, mobility, and temperature) for *spiddos P_i_* and *P^′^_i_* collected from two genome profiles (discriminated with or without a prime), respectively, and *i* denotes the serial number of *spiddos* [18].

GP needs to be carried out with the following specific precautions to get successful results. (i) During random PCR, contamination by other organisms should be carefully avoided. In particular, the entire random PCR solution without the template DNA should be UV-irradiated prior to the PCR reaction in order to inactivate any contaminating DNAs that could act as the template [12]. (ii) The random PCR reaction should be terminated before the primer molecules are consumed in order to attain a “double-strand stop,” which means that the major PCR products are in a double-stranded state and are suitable for TGGE analysis (i.e., the melting transition of double stranded DNA to single stranded one can be detected). (iii) The GP pattern should be strictly checked from the viewpoint of “quality score” in order to rule out false positives: the number of bands (usually more than eight are required) and the clearness of bands and background should be sufficient (note that random PCR generates DNA products at different molecular ratios (eventually, the sum of them is equivalent to that of the input primer) and, sometimes, overexpression of highly expressed DNAs (where the primer binds strongly for forward and reverse extension) suppresses the appearance of lower expressed ones, leading to less than eight observable bands). GP patterns that are sufficient in both the number and the clarity of bands are categorized “quality A” and used for the further analysis; those that are sufficient in only one of the two (but the other is still permissible) are categorized “quality B” and can be used with caution (note that quality B patterns were not used in this study); and the remaining patterns (“quality C”) should be discarded and the whole experiment should be retried.

### 2.4. Cluster analysis

We developed a clustering and displaying program termed “*Free Lighter*” on the basis of *Ward's method* [22, 23], which is a type of nearest neighbor method with an objective function of minimizing the “error sum of squares.” We also tested other derivative methods such as the *group average method*, *median method*, *furthest neighbor method*, as well as *Ward's method*, thereby confirming the well-known phenomenon of occasional minor changes in phylodendrons. These methods are based on the distance defined in (2) which implies that Clusters *a* and *b* are to be merged into *c*, and *x* is an arbitrary cluster:

(2) *d_c_* = *α_a_d_xa_* + *α_a_d_xb_* + *βd_xb_* + *γ*|*d_xa_d_xb_*|,

where *α_a_*, *α_b_*, *β_a_*, and *γ* are weighing parameters, *d_c_*, *d_xa_*, *d_xb_*, and *d_ab_* represent distances between relevant clusters such as Cluster *x* and Cluster *a* for *d_xa_*.

The parametric differences among these methods are *α_a_* = *n_a_*/*n_c_*, *α_b_* = *n_b_*/*n_c_*, *b* = 0, *γ* = 0 for the *group average method*; *α_a_* = 0.5, *α_b_* = 0.5, *β* = 0.25, *γ* = 0 for the *median method*; *α_a_* = 0.5, *α_b_* = 0.5, *β* = 0, *γ* = 0.5 for the *furthest neighbor method*; and *α_a_* = (*n_x_* + *n_a_*)/(*n_x_* + *n_c_*), *α_b_* = (*n_x_* + *n_b_*)/(*n_x_* + *n_c_*), *β* = *n_x_*/(*n_x_* + *n_c_*), *γ* = 0 for *Ward's method*. In this experiment, *d_ab_* was set to be 1-PaSS (*a, b*). The clustering results were found to be rather robust against changes in the above parameters, although there was a slight change in the order of neighbor joining in several cases.

## 3. RESULTS AND DISCUSSION

### 3.1. The method employed: genome profiling

The GP technology comprises three essential steps [13, 14, 18]. The first step is to extract DNA fragments specifically from the genomic DNA of an organism through random PCR [24], resulting in the specific reduction of the amount of DNA to be analyzed. The second step is *μ*TGGE to obtain the sequence-related information (i.e., a property related to the melting temperature of DNA [25]). The final step is to computer-process the experimental raw data (i.e., the band patterns in the gel) to obtain a normalized digital pattern (*spiddos*) that can be used for further analyses such as species identification and clustering [12, 18].

**Figure 1:**
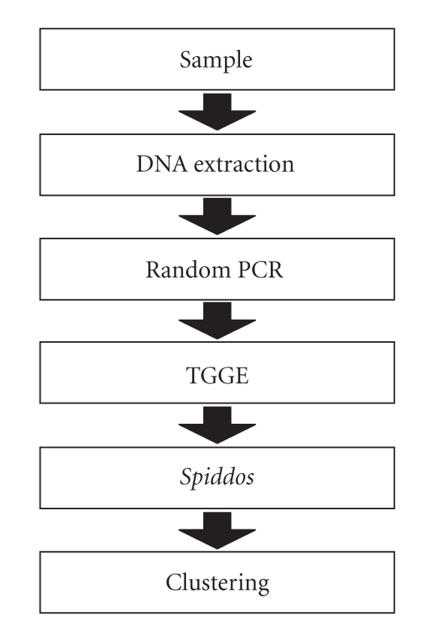
The procedure used to classify species by GP.

**Figure 2:**
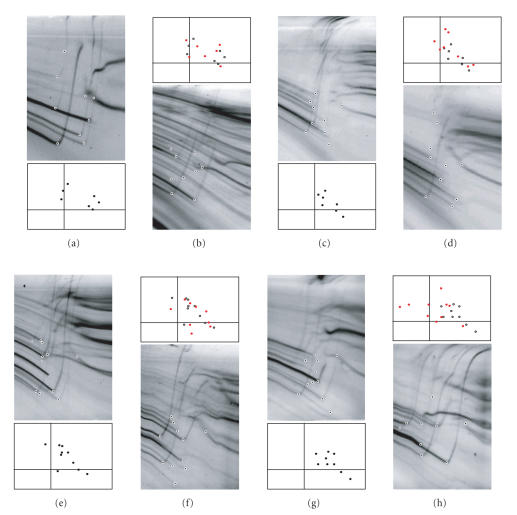
Genome profiles and *spiddos* patterns. Genome profiles of eight species of plant and fish are shown (top and bottom) together with their *spiddos* patterns (two Panels in the center): (a) Bulrush (A1); (b) Pansy (A9); (c) Dove tree (A11); (d) Hydrangea (A12); (e) Chinook salmon (C1); (f) Brown trout (C4); (g) European smelt (C8); (h) Stone loach (C12) (the same symbols as in Table 1 are used in the parenthesis). In the photographs, electrophoresis was performed from top to bottom with the temperature gradient running from left (low) to right (high temperature). The featuring points are plotted with a small black dot, whereas those corresponding to the internal reference DNA are plotted in white. *spiddos* (i.e., the normalized coordinates of featuring points) are dark in color (middle-upper panels) and red (middle-lower panels). For the comparison, blank *spiddos* (corresponding to those in the panel immediately above) are superimposed in each lower panel. The pairs a/c and e/f are relatively close, while the pairs c/d and g/h are relatively distant.

Figure 1 outlines the whole procedure used in this study to classify species by GP. Random PCR is a process in which DNA fragments are sampled at random from genomic DNA through the hybridization of a mismatch-containing primer to the template DNA during PCR [24]. In other words, this process is equivalent to the statistical approach used in random-sampling in a public opinion poll, from which an unbiased image of the whole can be grasped. *μ*TGGE [21] is used to get information related to the melting temperature (*T_m_*) of the sampled DNAs which is sequence-dependent [20]. Importantly, an internal reference DNA should be co-migrated on the gels in order to obtain experimental fluctuation-free (or normalized) data by the subtraction method [18]. Those featuring points that appear in electrophoretic band patterns (i.e., start of the melting transition; see Figure 2) are marked and converted to provide the coordinates of *spiddos* (species identification dots [18]).

By calculating a pattern similarity score (PaSS) between two genomes using *spiddos* as defined in Materials and Methods, we can get the information on how closely the two relevant genomes (organisms) are related (0 ≤ PaSS ≤ 1; PaSS = unity for a complete match). The PaSS value is known to be strongly correlated with the relatedness between two genomes although PaSS itself is based on a stochastic process [18]. This means that the random-sampling method cannot rule out the possibility of selecting a biased subpopulation; thus, the larger the sampling number of sampling becomes, the closer to the true image the sampled one will be. This is also the case with the GP approach; however, the sampling number of sampling can be increased by carrying out another random PCR using a different primer, resulting in the accumulation of information on a genome [13]. As far as the experiments that we have done are concerned (several hundreds of species and strains), a strong correlation between the PaSS value and the relatedness of two genomes has been observed [12, 18, 26], which can be theoretically rationalized [11] and is also experimentally verified in this paper (taking into consideration all of these facts, the PaSS value can be assumed to be semiquantitative as discussed later).

### 3.2. Three domains of organisms tested

**Figure 3:**
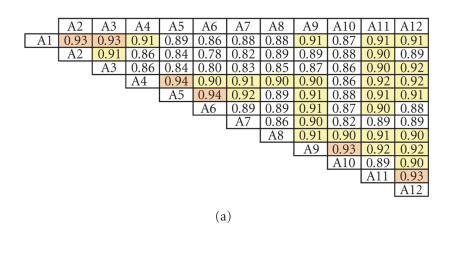

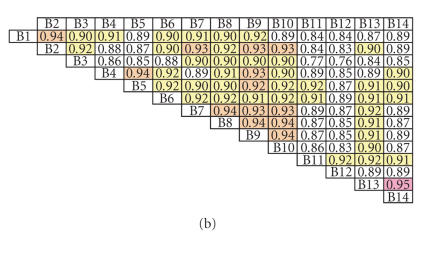

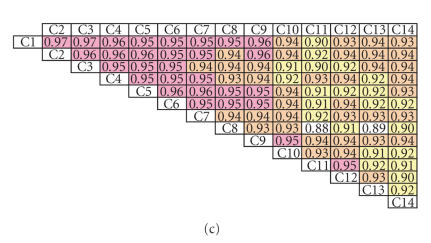
PaSS matrices for three domains of organisms. Colors represent the degree of PaSS values: Pink (≥0.95), Orange (<0.95 and ≥0.93), yellow (≤0.92 and ≥0.90), and white (<0.90).

**Figure 4:**
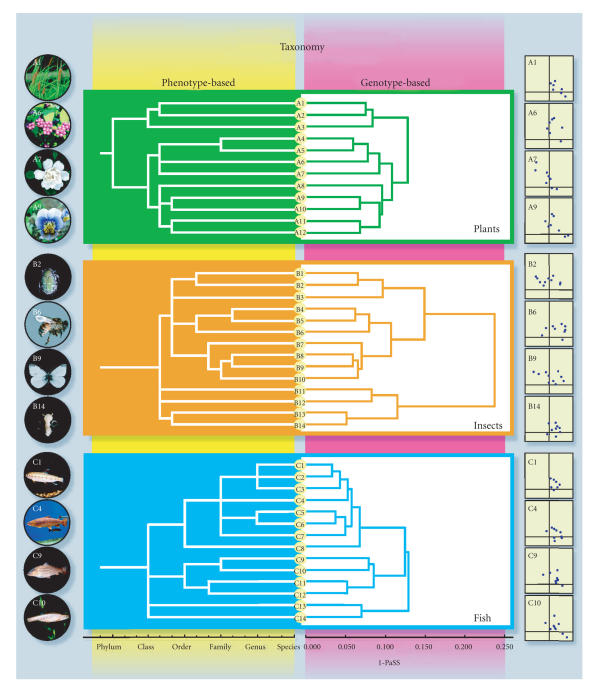
Phylodendrons of plants (A1 ∼ A12), insects (B1 ∼ B14), and fish (C1 ∼ C14). Phenotypic (left) and genotypic (right) trees are drawn on the basis of taxonomic hierarchy or PaSS value, respectively. The same nomenclature as in Table 1 are used. Photographs (far left) and *spiddos* (far right) are included to illustrate the technique. Trees were drawn by the *group average method* (plants) or the *median method* (insects and fish).

Here, to test our method, we collected and used three domains of organisms—namely, plants, fish, and insects—that are taxonomically well established (Table 1). Figures Figure 3(a)–Figure 3(c) shows the species that we tested here and the PaSS values obtained among them, respectively. To illustrate the technique, some of the results of GP and *spiddos* representations for plant and fish are shown in Figure 2, where the pairs of Panels a/b (A1: *Typha orientalis*, A9: *Viola xwittrockiana*) and Panels e/f (C1: *Oncorhynchus masou*, C4: *Salmo trutta*) apparently represent closer relationships than do the pairs of Panels c/d (A11: *Davidia involucrata*, A12: *Hydrangea macrophylla*) and g/h (C8: *Osmerus eperlanus mordax*, C12: *Barbatula barbatula*), as expected. The results obtained here for plants, fish, and insects were clustered (nearest neighbor method [27]) to determine intra-domains. On the basis of taxonomical knowledge, which has been established according to phenotypic traits, and the clustering results, two phylogenetic trees were constructed as shown in Figure 4. All of the organisms examined were classified topologically to the same position in both the phenotype- and the genotype-based trees (note that, throughout this study, no arbitrary selection of data was made except for ruling out a few low-quality data samples, meaning that the correspondence between the phenotype-based and the genotype-based classifications was perfect so far as tested).

### 3.3. Unexpected findings

It is surprising that such a limited amount of information (GP obtained with only a single oligonucleotide probe) can provide such perfect results for all organisms tested. Although it had been believed that GP should be able to classify species [28], it was considered that it would be better to use three or more probes per genome. Of course, another surprise is the strong correspondence between the results obtained by two quite different approaches: the traditional phenotype-based taxonomy (which by its nature is based on the well-considered but rather arbitrarily selected traits) and a genome sequence-based one (which is not directly based on sequence information itself). Theoretically, some ranks of hierarchy must be interconvertible in phenotype-based taxonomy so that such a correspondence is not a matter of course.

### 3.4. Methodological benefits

Apart from the rRNA and the other sequencing approaches described above, this is the first report describing the procedure of making phylogenetic trees of mutually-distant organisms based on the same criterion. Because the rRNA approach needs different pairs of primers to analyze a wide range of organisms, our simple approach is advantageous and can be used to complement the rRNA one. Notably, the length of branches in the phenotype-based tree is arbitrary and mainly implies topological meaning, whereas those in the genotype-based tree have a quantitative meaning: the longer the tree is, the more distantly related the organisms are. The results obtained here indicate that the quantitative expression of PaSS is very effective to some extent, even though the accuracy of the measure given by PaSS is thought to be limited, a priori, due to its stochastic nature [11] (i.e., there are some steps that are stochastic in nature and can influence determination of the PaSS value: for example, random PCR may or may not select a DNA fragment containing mutations, and the degree of displacement caused by a point mutation depends on the type of mutation such as A to G or A to C substitution [19], among others; further consideration will lead to the conclusion that this is the case not only with the GP approach but also with other approaches that depend on the comparison of a particular gene sequence [29]). In other words, even though the clustering of the organisms considered here was done, for the sake of simplicity, on the basis of a single experimental result obtained with a common oligonucleotide probe (dAGAACGCGCCTG, pfM12), the results were taxonomically consistent. At the current level of data (i.e., relatively small-scale sampling), we may be able to suggest conservatively that fish are widely classified on the basis of a more limited number of genes than are other organisms such as plants and insects, as can be seen in Figures 2 and 4, where relatively small differences in the genome sequence (measured by PaSS) are observed among species of fish, although the possibility of biased sampling cannot be completely ruled out.

### 3.5. Expandable amount of information

Evidently, by using more kinds of probes, taxonomically more reliable results can be obtained, as we have demonstrated experimentally for other organisms such as fungi and rice (to be published elsewhere). An excellent feature of this methodology is that the amount of information used for classification can be increased on demand [18] without limitation, simply by repeatedly performing an additional random PCR with a different oligonucleotide probe and thus obtaining additional *spiddos* (which can be expressed as “*information-scalable*”). Thus, this methodology has the potential to become a highly accurate classification tool, as well as a convenient, universal one. Moreover, it has been already demonstrated that DNAs that provide *spiddos* can be collected and sequenced if necessary [26, 30].

As mentioned above, GP has proved to be useful for identifying species [12, 14], in addition to classifying species as shown here. It could therefore be said that the ability of GP to classify species was indirectly supported by the earlier studies, but it had not been explicitly demonstrated. We cannot but feel the splendor of the correspondence between the phenotypic world and the genotypic one, although its meaning needs to be considered more deeply hereafter.

## Figures and Tables

**Table 1:** Taxonomy^†^ of the species dealt with in this study.

No.	Species/conventional name	Family	Order	Calss	Phylum

A1	*Typha orientalis*/Bulrush sp.	Typhaceae	Typhales	Mono*	Anth*
A2	*Arundinaria argenteostriata*/Bamboo sp.	Poaceae	Cyperales	Mono*	Anth*
A3	*Tricyrtis hirta*/Lily sp.	Liliaceae	Liliales	Mono*	Anth*
A4	*Cosmos bipinnatus*/Cosmos sp.	Asteraceae	Asterales	Dico*	Anth*
A5	*Taraxacum officinale*/Dandelion sp.	Asteraceae	Asterales	Dico*	Anth*
A6	*Callicarpa dichotoma*/Beauty-berry sp.	Verbenaceae	Lamiales	Dico*	Anth*
A7	*Gardenia jasminoides*/Gardenia sp.	Rubiaceae	Rubiales	Dico*	Anth*
A8	*Papaver nudicaule*/Poppy sp.	Papaveraceae	Papaverales	Dico*	Anth*
A9	*Viola xwittrockiana*/Pansy sp.	Violaceae	Violales	Dico*	Anth*
A10	*Camellia sasanqua*/Camellia sp.	Theaceae	Theales	Dico*	Anth*
A11	*Davidia involucrata*/Dove tree sp.	Davidiaceae	Cornales	Dico*	Anth*
A12	*Hydrangea macrophylla*/Hydrangea sp.	Hydrangeaceae	Rosales	Dico*	Anth*

B1	*Chilocorus rubidus*/Beetle sp. 1	Coccinellidae	Coleoptera	Inse*	Arth*
B2	*Oxycetonia jucunda*/Beetle sp. 2	Scarabaeidae	Coleoptera	Inse*	Arth*
B3	*Bombylius major*/Horse fly sp.	Bombyliidae	Diptera	Inse*	Arth*
B4	*Camponotus japonicus*/Ant sp. 1	Formicidae	Hymenoptera	Inse*	Arth*
B5	*Formica japonica*/Ant sp. 2	Formicidae	Hymenoptera	Inse*	Arth*
B6	*Apis mellifera*/Bee sp.	Apidae	Hymenoptera	Inse*	Arth*
B7	*Limenitis camilla*/Butterfly sp. 1	Nymphalidae	Lepidoptera	Inse*	Arth*
B8	*Anthocharis scolymus*/Butterfly sp. 2	Pieridae	Lepidoptera	Inse*	Arth*
B9	*Pieris rapae crucivora*/Butterfly sp. 3	Pieridae	Lepidoptera	Inse*	Arth*
B10	*Eurema laeta*/Butterfly sp. 4	Pieridae	Lepidoptera	Inse*	Arth*
B11	*Gonolabis marginalis*/Earwig sp.	Anisolabididae	Dermaptera	Inse*	Arth*
B12	*Bothrogonia ferruginea*/Stinkbug sp.	Cicadellidae	Hemiptera	Inse*	Arth*
B13	*Blattella germanica*/Cockroach sp.	Blattellidae	Blattaria	Inse*	Arth*
B14	*Reticulitermes speratus*/Termite sp.	Rhinotermitidae	Isoptera	Inse*	Arth*

C1	*Oncorhynchus masou*/Salmon sp. 1	Salmonidae	Salmoniformes	Acti*	Chor*
C2	*Oncorhynchus tshawytscha*/Salmon sp. 2	Salmonidae	Salmoniformes	Acti*	Chor*
C3	*Oncorhynchus mykiss*/Rainbow trout	Salmonidae	Salmoniformes	Acti*	Chor*
C4	*Salmo trutta*/Brown trout	Salmonidae	Salmoniformes	Acti*	Chor*
C5	*Salvelinus malma malma*/Dolly Varden	Salmonidae	Salmoniformes	Acti*	Chor*
C6	*Salvelinus leucomaenis*/Whitespotted char	Salmonidae	Salmoniformes	Acti*	Chor*
C7	*Hucho perryi*/Japanese huchen	Salmonidae	Salmoniformes	Acti*	Chor*
C8	*Osmerus eperlanus mordax*/Rainbow smelt	Osmeridae	Salmoniformes	Acti*	Chor*
C9	*Cyprinus carpio*/Carp sp. 1	Cyprinidae	Cypriniformes	Acti*	Chor*
C10	*Phoxinus percnurus*/Carp sp. 2	Cyprinidae	Cypriniformes	Acti*	Chor*
C11	*Misgurnus anguillicaudatus*/loach sp. 1	Cobitidae	Cypriniformes	Acti*	Chor*
C12	*Barbatula barbatula*/loach sp. 2	Balitoridae	Cypriniformes	Acti*	Chor*
C13	*Silurus asotus*/Amur cat fish sp.	Siluridae	Siluriformes	Acti*	Chor*
C14	*Cottus nozawae*/Bullhead sp.	Cottidae	Scorpaeniformes	Acti*	Chor*

^†^This table is built based on NCBI's Taxonomy (http://www.ncbi.nlm.nih.gov/entrez/query.fcgi? CMD = search & DB = taxonomy) and Iwanami Biology Encyclopedia, 4th edition [15].

*Mono: Monocotyledonopsida, Dico: Dicotyledonopsida, Anth: Anthophyta, Inse: Insecta, Acti: Actinopterygii, Chor: Chordata.
